# Book Review

**DOI:** 10.1155/S146392468100045X

**Published:** 1981


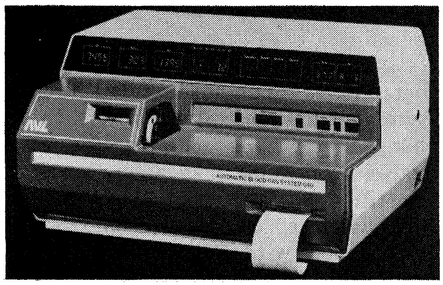

*The AVL 940 automatic blood gas analyser from Laboratory Impex*

### Small sample blood gas analyser

A fully automated pH/blood gas analyser working on 40 µl samples is now available in the UK from Laboratory Impex. The AVL 940 produces digital and hard copy records of $PO_2$, $PCO_2$ and pH. $PO_2$ and $PCO_2$ concentrations are displayed in mmHg, or a readout in kPa is available. The instrument then requires type of patient (adult or foetal), patient temperature and Hb level to provide the following calculated values: base excess *in vivo* and *in vitro*, buffer base, actual bicarbonate, total $CO_2$, oxygen saturation and oxygen content. Full analysis takes 50 seconds plus 15 seconds for presentation of results.

*Laboratory Impex, Lion Rd, Twickenham, Middx. Tel 01 892 9157*

### Recording data processor

Shimadzu has introduced a computerised data processor for use with GC, LC, TLC and AA. The machine features continuously variable peak width integration, full battery protected memory and alpha numeric capability. Up to 339 peaks can be stored and processed to provide data identifications using either absolute retention time, relative retention time, time band or time window methods. Quantitation methods such as area normalisation, corrected area normalisation, scale factors, internal standards and absolute calibration curve are available and concentrations can be presented in any unit of percent, mg, µg and ppm.

*Shimadzu Scientific Instruments Inc, 9147-H Red Branch Rd, Columbia, MD 21045, USA*

### Publications

Varian has produced a 70 page buyers' guide presenting its entire **liquid chromatography** range from simple isocrate devices to the fully automatic LC system. There is also a synopsis of various publications and audio-visual aids on the basics of LC.

*Varian Associates Ltd, 24-28 Manor Rd, Walton-on-Thames, Surrey*

The second quarter, 1981 issue of Technicon's New Products Newsletter, has information on the company's updated product lines, including the new GT system for **accelerated throughput on the AutoAnalyser II**, introduced at Pittsburg, and the AutoAnalyser IIC Plus system for data handling and instrument control.

*Technicon Industrial Systems, Tarrytown, New York 10591 USA*

From Techmation comes a catalogue describing laboratory, on-line and field instrumentation for the analysis and monitoring of principal **air and water pollutants** and physical measurements. Selected products from ten manufacturers are presented.

*Techmation Ltd, 58 Edgware Way, Edgware, Middx. Tel 01 958 3111*

The complete range of Camag **TLC instruments** is presented in a 48 page brochure now available from the company. All phases of TLC process are covered from sample application up to quantitative (densitometric) evaluation. Detail descriptions of apparatus are accompanied by methodological explanations.

*Available from any Camag distributor or agent*

### Book Review

### Electronics and instrumentation for scientists

*Howard V. Malmstadt, Christie G. Enke and Stanley R. Crouch*
*The Benjamin/Cummings Publishing Company Inc, USA*
*Order code: 36917. Price £24.95*

This is the latest edition of what has become one of the standard works for the instruction of scientists, and particularly chemists, in basic electronics and instrumentation. The aim of the book is to provide scientists with an efficient means of understanding the principles of modern electronic instrumentation so that they can confidently choose instruments and apply them to their full potential. The subject is therefore modern electronics with an emphasis on its applications in scientific instrument and control systems and computers. In all its editions the book has been written particularly for students and practitioners in all areas of science and engineering. The presentation is both practical and fundamental. The new edition of the book reflects the fact that we have now entered a new era in scientific instrumentation; the era of microelectronics. Microcircuits provide both the basis and the need for a new approach to the study of electronics. From the users point of view, the availability of complete high performance electronic functions such as operational amplifiers and decade counters in encapsulated, unalterable integrated circuit form, makes the detailed study of transistor amplifier and flip-flop design less critical than a knowledge of the functions performed by the IC's and their applications. At the same time the widespread application of sophisticated electronic functions such as phase-locked loops, analog multipliers and microprocessors creates the need to understand the principles of advanced measurement and control techniques that are being used in the most common laboratory instruments.

The book is organised around system principles and electronic functions rather than the detailed analysis of particular components. The latter however are not neglected, since components of all types, from resistors through transistors and integrated circuits, are studied as the devices by which the various functions can be implemented. Because of this approach, new devices will not make obsolete the knowledge gained from this study; rather they will be recognised as new ways to achieve the appropriate functions. Analog and digital techniques are integrated through the concept of electrical data domains, which are the ways in which data can be encoded in electrical signals. Instrumentation concepts including feedback control, signal-to-noise enhancement, microcomputer interfacing, data acquisition and signal processing are presented along with the techniques and devices for their implementation.

The book as in its earlier editions is useful for structured coursework or for self study. The fourteen chapters included are of nearly uniform length and complexity so that each comprises a similar unit of study. The problems at the end of each chapter are useful for review. This book is organised to support a parallel laboratory if structured coursework is concerned. A list of suggested experiments follows each chapter to indicate the areas of practice the chapter has introduced.

The authors are to be congratulated on the new edition of this standard work; the high standard of production seen in earlier editions has been maintained and its value has been increased immeasurably by the revisions made to include more of the microelectronics which are pertinent to modern analytical instrumentation.

*G.F. Kirkbright*

